# Fentanyl activates ovarian cancer and alleviates chemotherapy-induced toxicity via opioid receptor-dependent activation of EGFR

**DOI:** 10.1186/s12871-022-01812-4

**Published:** 2022-08-23

**Authors:** Kai Xiao, Qinghong Zheng, Lei Bao

**Affiliations:** Department of Anesthesia, Wuhan Fourth Hospital, 473 Hanzheng Street, Qiaokou District, Wuhan, 430033 Hubei China

**Keywords:** Fentanyl, µ opioid receptor, EGFR, Ovarian cancer, Antagonism

## Abstract

**Background:**

Fentanyl is an opioid analgesic and is widely used in ovarian cancer patients for pain management. Although increasing evidence has suggested the direct role of fentanyl on cancer, little is known on the effect of fentanyl on ovarian cancer cells.

**Methods:**

Proliferation, migration and apoptosis assays were performed in ovarian cancer cells after fentanyl treatment. Xenograft mouse model was generated to investigate the in vivo efficacy of fentanyl. Combination index was analyzed for the combination of fentanyl and chemotherapeutic drugs. Immunoblotting approach was used to analyze signaling involved in fentanyl’s action focusing on EGFR.

**Results:**

Fentanyl at nanomolar concentration does-dependently increased migration and proliferation of a panel of ovarian cancer cell lines. Fentanyl at the same concentrations either did not or stimulated proliferation to a less extent in normal cells than in ovarian cancer cells. Consistently, fentanyl significantly promoted ovarian cancer growth *in vivo*. The combination of fentanyl with cisplatin or paclitaxel was antagonist in inhibiting cell proliferation. Although fentanyl did not affect cell apoptosis, it significantly alleviated ovarian cancer cell death induced by chemotherapeutic drugs. Mechanistically, fentanyl specifically activated EGFR and its-mediated downstream pathways. Knockdown of EGFR abolished the stimulatory effects of fentanyl on ovarian cancer cells. We finally demonstrated that the activation of EGFR by fentanyl is associated with opioid µ receptor system.

**Conclusions:**

Fentanyl activates ovarian cancer via simulating EGFR signaling pathways in an opioid µ receptor-dependent manner. The activation of EGFR signaling by fentanyl may provide a new guide in clinical use of fentanyl in ovarian cancer patients.

**Supplementary Information:**

The online version contains supplementary material available at 10.1186/s12871-022-01812-4.

## Background

Epithelial ovarian cancer, accounts for 90% of ovarian malignancies, is the most frequent cause of gynecologic cancer death worldwide [1]. Conventional treatment includes cytoreductive surgery followed by platinum and taxane chemotherapy [2]. Cisplatin and paclitaxel are the first line chemotherapeutic drugs for ovarian cancer. However, majority of patients develop chemo-resistance and recurrence [3]. Once ovarian cancer recurs, all subsequent treatments are palliative. Aberrant epidermal growth factor receptor (EGFR) is found in ~ 60% of ovarian cancers and is correlated with poor prognosis, drug resistance, metastasis and low survival rate [4]. The activated EGFR stimulates the activation of various intracellular signaling pathways that play essential roles in cancer cell proliferation, migration and survival, including the Ras/Raf/Mek/ERK, PI3K/Akt, STAT and Src pathways [5]. In patients with advanced ovarian cancer, opioids are constantly applied for the clinical management of pain [6]. Opioids act on central nervous system via binding to opioid receptors which are members of the G protein coupled receptor (GPCR) superfamily and are classified as μ, δ and κ [7].

Morphine, fentanyl and oxycodone that are frequently used for the palliative care of cancer patients are mainly μ-opioid receptor agonists [8]. Recent studies revealed that morphine and oxycodone can activate EGFR and stimulate EGFR-mediated signaling pathways in cancer cells [9, 10]. Compared to morphine and oxycodone, fentanyl is a synthetic opioid with stronger analgesic effect and less adverse effect [11]. Substantial pre-clinical evidence has shown that fentanyl displays direct effects on cancer cells. Fentanyl inhibits proliferation and invasion of colorectal cancer [12, 13], and inhibits pancreatic cancer cell proliferation and cancer stem cell differentiation [14]. Interestingly, fentanyl also promotes breast cancer cell stemness and induces epithelial-mesenchymal transition [15]. The effect of fentanyl on ovarian cancer is unknown.

Given the importance of EGFR in ovarian cancer and the fact that μ-opioid receptor agonists can activate EGFR, we hypothesized that fentanyl might display pro-ovarian cancer activity via simulating EGFR-mediated signaling. We investigated the effect of fentanyl on ovarian cancer cell growth, migration and survival, and its combinatory effects with chemotherapeutic drugs. In addition, we examined the effect of fentanyl on EGFR-mediated signaling and its association with μ-opioid receptor in ovarian cancer.

## Methods

### Cell lines and drug treatment

The three human ovarian cancer cell lines were obtained from the Cell Bank of Type Culture Collection of Chinese Academy of Sciences. Normal human immortalized ovarian cell line was purchased from abm Inc. Cells were sub-cultured using RPMI 1640 medium, containing 10% fetal bovine serum (Gibco) and 1% penicillin/streptomycin (Sigma), in 37^0^C, 5% CO_2_ atmosphere. Cells in exponential growth phase were used in this study. Fentanyl (Yichang Humanwell Pharmaceutical), paclitaxel and cisplatin (Sigma) were reconstituted in dimethyl sulfoxide (DMSO) and saline (0.9% NaCl w/w), respectively. Cells were incubated with drugs for 3 days for proliferation and apoptosis assays; 12 h for migration assay; and 24 h for western blot analysis.

### Proliferation assay and combination index (CI) calculation

Proliferating cells were determined using Bromodeoxyuridine/5-bromo-2'-deoxyuridine (BrdU) proliferation assay kit (Abcam). After drug treatment, BrdU was then added to the cell medium. The dividing cells were labelled with BrdU, were fixed with fixing solution, and were detected using an anti-BrdU antibody. The quantity of BrdU incorporation was measured using microplate reader via reading absorbance at 450 nm. Combination studies were designed based on Chou and Talalay method and combination index was calculated using CalcuSyn software [16]. Briefly, cells were treated with increasing doses of single drug alone, or the combination of both with an equipotent constant-ratio. Proliferation was determined and CI was determined after the data entries based on dose and effect of single drug and combinations.

### Apoptosis assay

Apoptotic cells were determined using Cell Death Detection ELISA kit (Roche). After drug treatment, cells were detached using trypsin (Sigma) and resuspended in lysis buffer provided by the kit. Cytoplasmic histone-associated DNA fragments were detected using anti-histone biotin and quantified as per manufacture’s protocol.

### Boyden Chamber migration assay

Migration assay was performed using the Boyden chamber (Cell Biolabs) as described in our previous studies [17, 18]. Briefly, 1000 cells were added onto upper chamber, and drugs were added onto the lower chamber. After 12 h incubation, the non-migratory cells on the upper surface of the insert were removed with cotton bud. Migratory cells on the lower surface of inserts were fixed with 4% formaldehyde (Sigma) and stained with 0.4% Giemsa. The photos were taken under microscope and migrated cells from five fields (up, low, left, right and centre) were counted for quantification.

### siRNA knockdown

Knockdown of EGFR was performed using siRNA. The target sequencing of si-EGFR1 and si-EGFR2 are 5’-UGA UCU GUC ACC ACA UAA UUA CGG-3’ and 5’-UUA GAU AAG ACU GCU AAG GCA UAGG-3’. Cells were seeded in a 6 -well plate at 70% confluency, and were transfected with 100 nM siRNA using Lipofectamine 3000 transfection reagent (Invitrogen) as per the manufacture’s protocol. Cells were collected for protein expression analysis at 72 h post-transfection.

### Western blot (WB) analyses

Drug treated cells were lysed for total protein extraction using radioimmunoprecipitation assay buffer (Invitrogen). Protein concentrations were measured using QuantiPro BCA Assay kit (Invitrogen). Equal amount of proteins was loaded onto denaturing sodium dodecyl sulfate–polyacrylamide gel (SDS-PAGE) and resolved via electrophoresis. Protein were then transferred onto polyvinylidene fluoride membranes (Bio-Rad), followed by WB analysis using standard protocol. The blots were cut prior to hybridisation with antibodies during blotting. All antibodies were purchased from Abcam. Signal was developed using enhanced chemiluminescent reagent (Pierce).

### Ovarian cancer growth in mice

Severe combined immunodeficiency (SCID) mice were purchased from Hunan SJA Laboratory Animal Co., Ltd and were housed in a pathogen-free environment. SK-OV-3 cells were resuspended in 100 µl PBS and were subcutaneously injected into mice flank. After development of palpable tumors, mice were treated with citrate buffer as vehicle control or fentanyl at 10, 20 and 40 ng/kg once per day. Fentanyl was subcutaneously injected into tumor surrounding site. Mice body weight were measured once per week. Tumor volumn was calculated using the formula: length x width^2^ /2. Once tumor size exceeded 1500 mm^3^, mice were euthanized using CO_2_ inhalation.

### Statistical analysis

All data were obtained from at least three independent experiments and expressed as mean ± SD. T-test and analysis of variance (ANOVA) was used for statistical analysis. *P* < 0.01 was considered as statistical significance.

## Results

### Fentanyl activates ovarian cancer cell migration and growth *in vitro* and *in vivo*

**Fig. 1:**
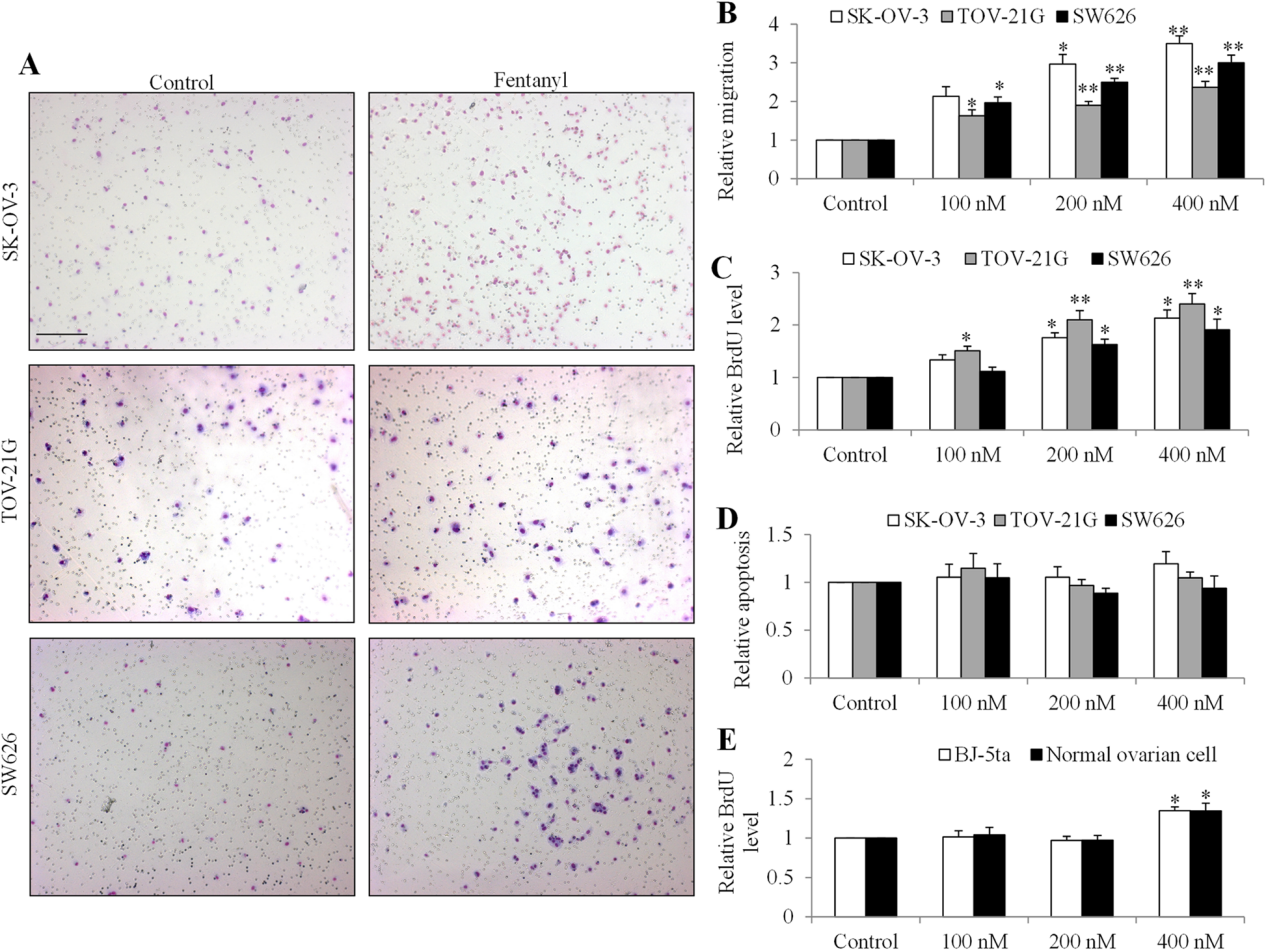
Fentanyl stimulates ovarian cancer biological activities. Fentanyl (**A**) Representative images of Chamber Boyden migration using three different ovarian cancer cell lines in the absence or presence of fentanyl (400 nM). Photos of the migrated cells on the center of field were taken under light microscope. Scale bar is 20 µm. Fentanyl at 100, 200, and 400 nM significantly increases ovarian cancer cell migration (**B**) and proliferation (**C**) in a dose-dependent manner. **D** Fentanyl up to 400 nM does not affect ovarian cancer apoptosis. **E** Fentanyl at 400 nM but not 100 or 200 nM significantly increases BJ-5ta and normal ovarian cell proliferation. Two types of normal cell lines were used. One is BJ-5ta which is a human fibroblast cell line. Another is Immortalized Human Ovarian Epithelial Cells—SV40. *, *P* < 0.01; **, *P* < 0.001, compared to control

**Fig. 2:**
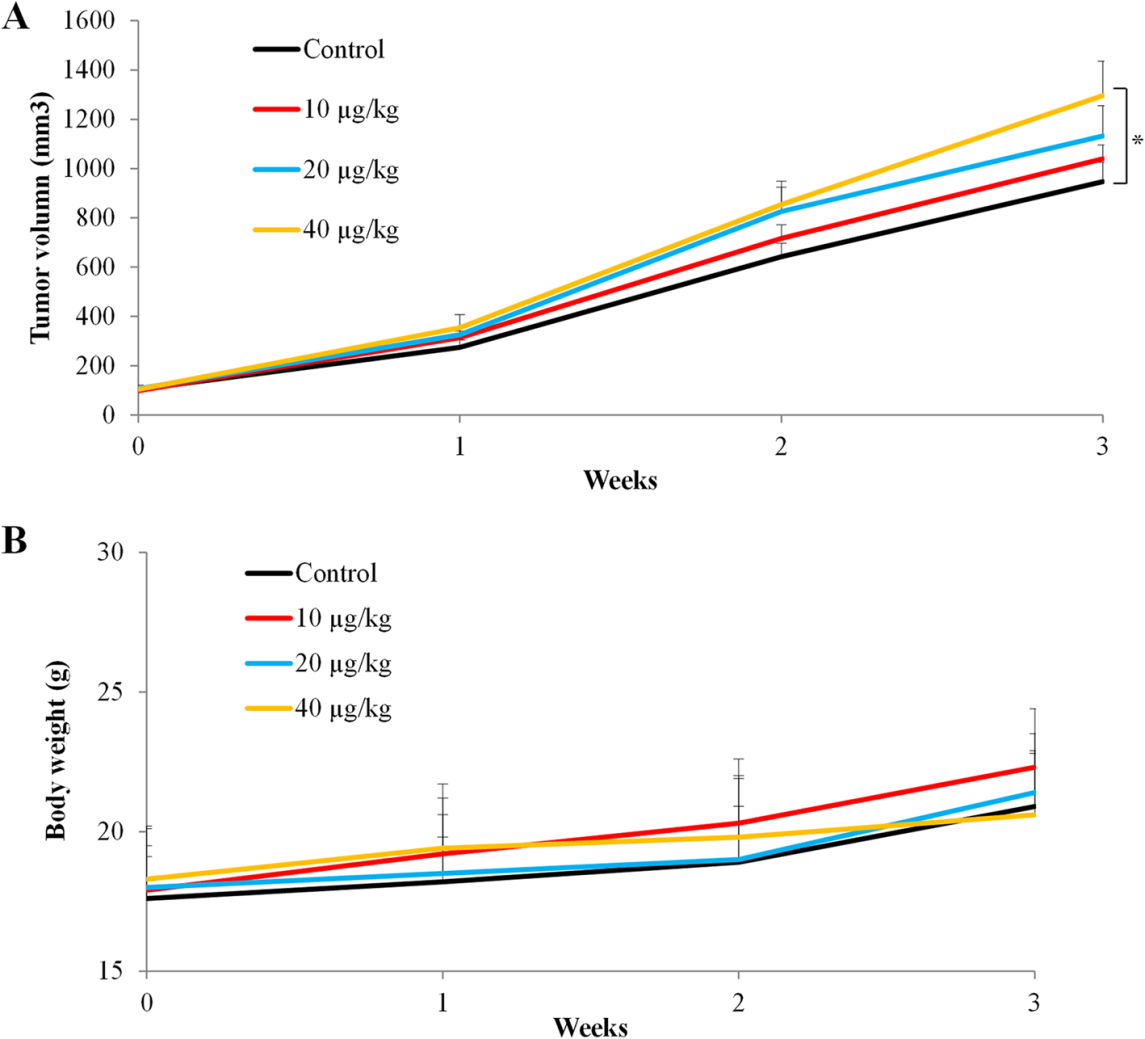
Fentanyl significantly stimulates ovarian cancer growth in mice. **A** Fentanyl at 40 ng/kg but not 10 ng/kg or 20 ng/kg significantly promoted ovarian cancer growth in mice. **B** Mice body weight at different drug treatment groups. *p < 0.05, compared to control

To investigate whether fentanyl affects ovarian cancer cell migration, proliferation and survival, we performed Boyden Chamber migration assay, measured BrdU level and cytoplasmic histone-associated DNA fragments using three human ovarian cancer cell lines with different cellular resources and genetic profiling. As shown in Fig. 1A, we observed the increased number of migrated cells in the presence of fentanyl. Quantification showed that fentanyl at nanomolar concentrations significantly increased migration in a dose-dependent manner by up to 2.3- to 3.5- fold in all tested ovarian cancer cell lines: SK-OV-3, TOV-21G and SW626 (Fig. 1B and Table S1, *p* < 0.001). We also found that fentanyl at the same concentrations significantly increased ovarian cancer growth by up to 1.9- to 2.4- fold as measured by BrdU level (Fig. 1C and Table S2, *p* < 0.01). The degradation and compaction of histones are hallmark features of apoptosis [19]. To investigate whether fentanyl affects ovarian cancer cell apoptosis, we assessed cytoplasmic histone-associated DNA fragments after fentanyl treatment. We found that fentanyl at 100, 200 and 400 nM did not affect the level of cytoplasmic histone-associated DNA fragments (Fig. 1D and Table S3), suggesting that fentanyl does not affect apoptosis. Taken together, these results demonstrate that fentanyl stimulates ovarian cancer cell growth and migration without affecting apoptosis. We performed proliferation assay on two normal cell lines (human immortalized ovarian epithelial cell line and human fibroblast cell line BJ-5ta) under the same experimental conditions. We found that fentanyl at the same concentrations either did not (100 nM and 200 nM) or stimulated (400 nM) proliferation to a less extent in normal cells than in ovarian cancer cells (Fig. 1E). To demonstrate the *in vivo* efficacy of fentanyl in ovarian cancer, we generated ovarian cancer model by subcutaneously injecting SK-OV-3 cells in SCID mice and tested three doses of fentanyl. We found that fentanyl at 40 ng/kg but not 10 or 20 ng/kg significantly promoted tumor growth by ~ 1.5-fold compared to control (Fig. 2A). In addition, the mice body weight was not significantly changed in all drug treatment groups (Fig. 2B), suggesting that fentanyl at effective dose is not toxic in mice.

### Fentanyl reverses the anti-proliferative and pro-apoptotic effects of chemotherapeutic drugs in ovarian cancer cells

**Fig. 3:**
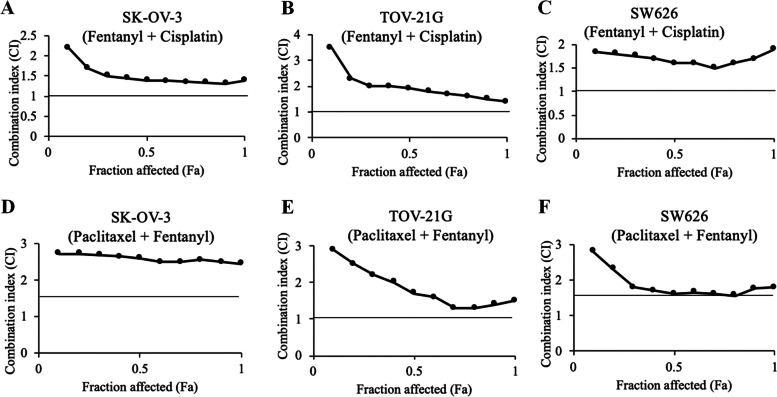
The combination of fentanyl with chemotherapeutic drugs is antagonistic in ovarian cancer cells. Isobologram analysis of combination index (CI) values of the combination of fentanyl with cisplatin or paclitaxel in all fractions are more than 1 in SK-OV-3 (**A** and **D**), TOV-21G (**B** and **E**) and SW626 (**C** and **F**) cells. CI was calculated using the Calcusyn software. CI of less than 1 indicates synergism; CI equals to 1 indicates additivity; and CI of greater than 1 indicates antagonism of the two drugs in combination

**Fig. 4:**
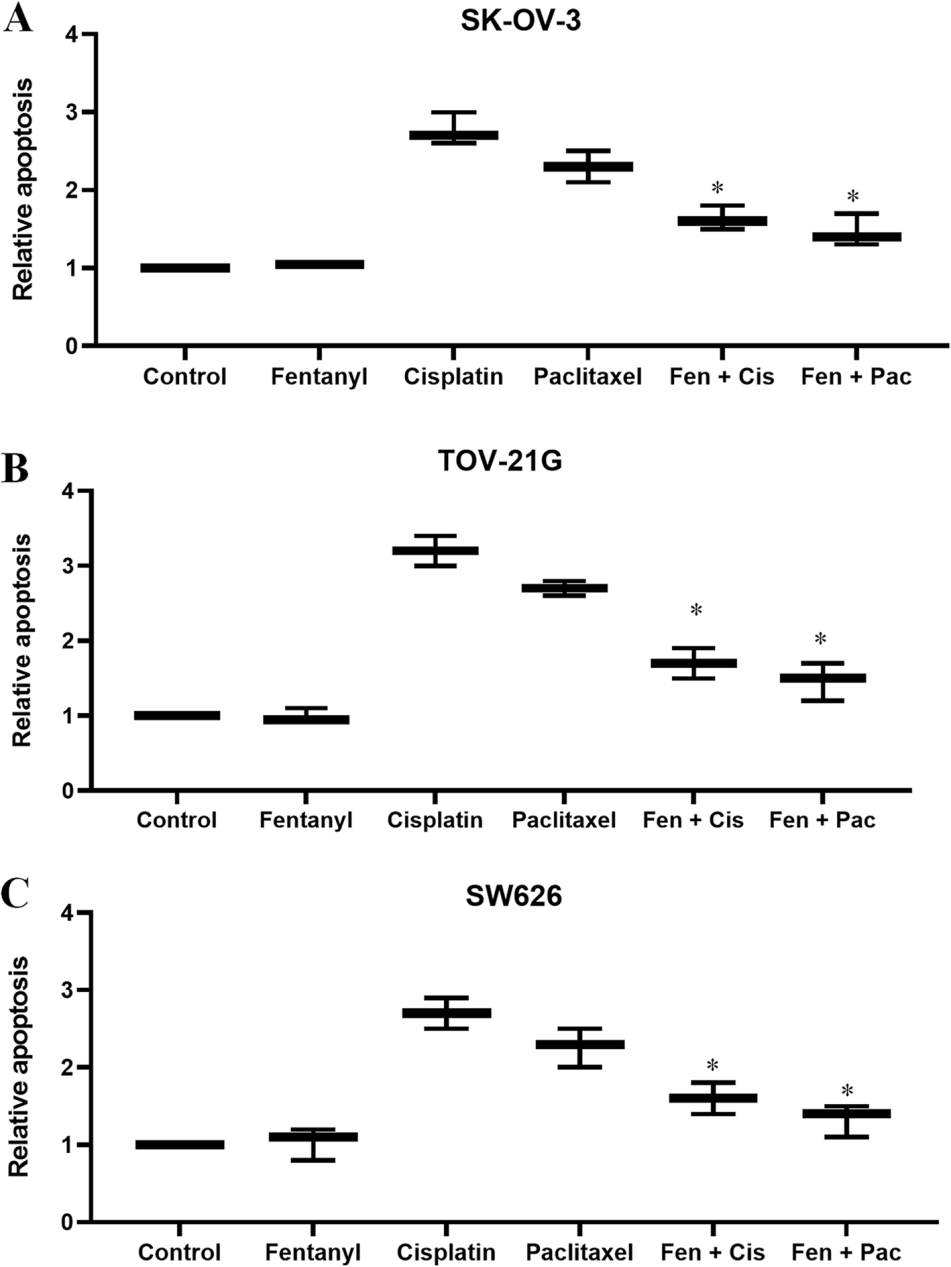
Fentanyl reveres the pro-apoptotic effect of chemotherapeutic drugs in ovarian cancer cells. The combination of fentanyl (400 nM) with cisplatin (5 µM) or paclitaxel (100 nM) induced significantly less apoptosis than cisplatin or paclitaxel alone in SK-OV-3 (**A**), TOV-21G (**B**) and SW626 (**C**) cells. *, *P* < 0.01, compared to cisplatin or paclitaxel

To examine whether fentanyl protects ovarian cancer cells against chemotherapeutic drugs-induced cytotoxicity, we designed combination studies based on Chou and Talalay’s method and calculated combination index (CI) to determine whether the combination is synergistic (CI < 1), additive (CI = 1) or antagonistic (CI > 1) [16]. Both cisplatin and paclitaxel which are the common therapeutic drugs for the treatment of ovarian cancer were tested in the combination studies. CI of fentanyl and cisplatin combination at 0–100% growth inhibition are less than 1 (Fig. 3A to C), demonstrating that the combination of fentanyl and cisplatin is antagonistic in inhibiting ovarian cancer cell proliferation. The antagonism was also observed between fentanyl and paclitaxel (Fig. 3D to F). Of note, although fentanyl alone does not affect ovarian cancer cell apoptosis, the combination of fentanyl with cisplatin or paclitaxel induced significantly less apoptosis than cisplatin or paclitaxel alone (Fig. 4 and Table S4, *p* < 0.001). This is consistent with the findings of combination studies, demonstrating that the addition of fentanyl reveres the pro-apoptotic effect of chemotherapeutic drugs in ovarian cancer cells.

### Fentanyl activates EGFR-mediated pathways and induces mesenchymal-epithelial transition (EMT) in ovarian cancer cells

**Fig. 5:**
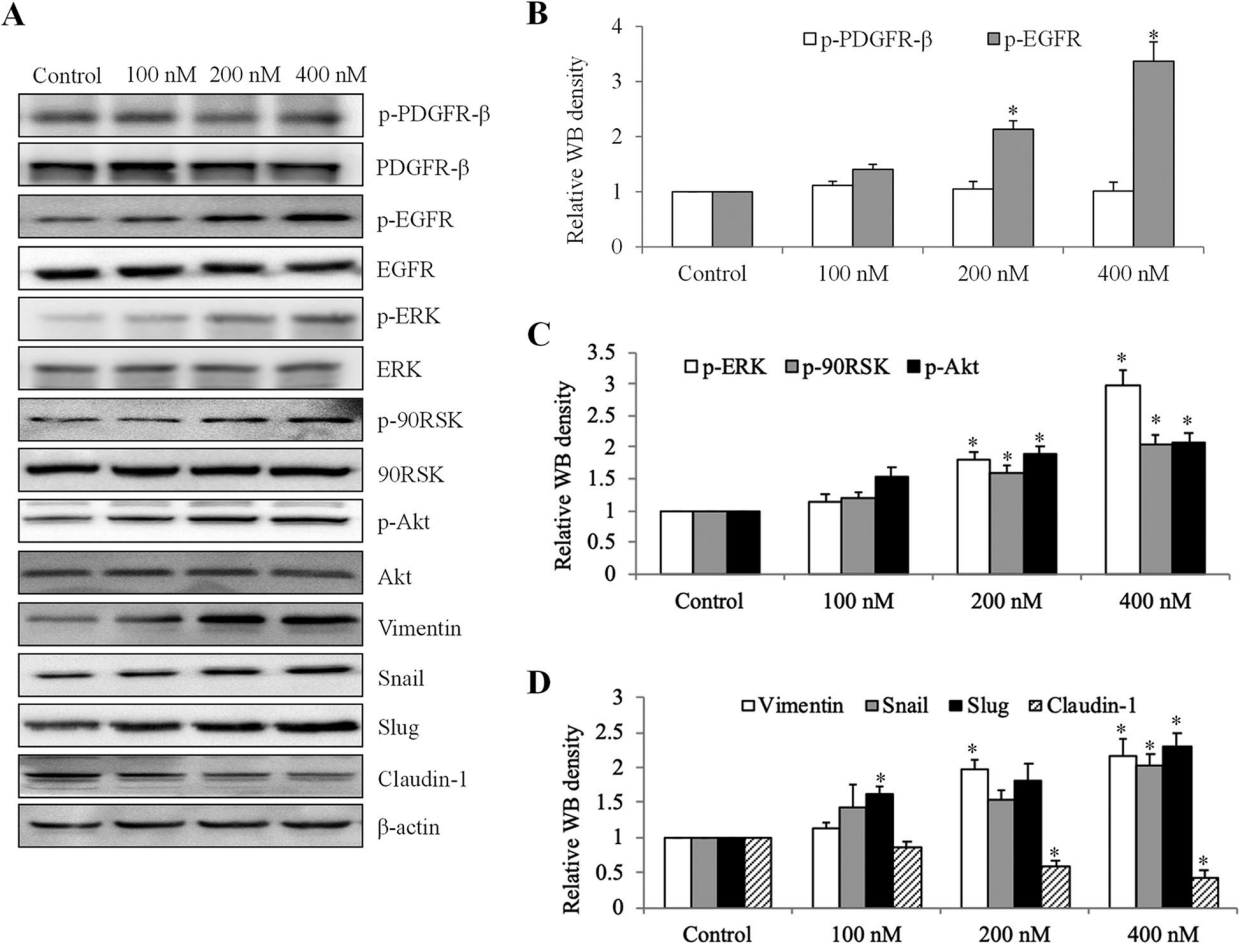
Fentanyl activates EGFR-mediated signaling and increases epithelial mesenchymal transition (EMT) in ovarian cancer cells. **A** Representative western blot of SK-OV-3 cells after 24 h exposure of fentanyl. Antibodies used in western blot analyses include anti-p-EGFR (Tyr1173), anti-EGFR, anti-p-90RSK (T359/S363), anti-90RSK, anti-p-PDGFR-β (T857), anti-PDGFR-β, anti-p-ERK (T202/Y204), anti-ERK, anti-Akt (S473), anti-Akt, anti-Vimentin, anti-Snail, anti-Slug and anti-Claudin-1. The blots were cut prior to hybridization with antibodies during blotting. **B** Fentanyl significantly increases p-EGFR but not p-Src in ovarian cancer cells. **C** Fentanyl significantly increases p-ERK, p-90RSK and p-Akt. **D** Fentanyl significantly increases Vimentin, Snail and Slug, and decreases Claudin-1. *, *P* < 0.01, compared to control

The direct pro-cancer molecular targets of fentanyl have rarely been elucidated. Several μ-opioid receptor agonists, such as morphine and oxycodone, have been shown to activate EGFR signalling [9, 10]. Given the importance of EGFR activation in ovarian cancer [4], we firstly investigated the EGFR signalling using immunoblotting approach in fentanyl-treated ovarian cancer cells. As shown in Fig. 5A and B and Fig. S1, fentanyl treatment resulted in significantly increased phosphorylation of EGFR by 3.4-fold but not placental derived growth factor receptor (PDGFR), demonstrating the specific activation of EGFR by fentanyl in ovarian cancer cells. We further observed a dose-dependent increase in p-ERK, p-90RSK and p-Akt by up to 2- to threefold in fentanyl-treated ovarian cancer cells (Fig. 5A and C, Table S5, *p* < 0.001), suggesting the activation of MEK/ERK and PI3K/Akt which are the major downstream signaling pathways of EGFR [20]. Consistent with Lennon et al.’s work [21], we observed the increased level of vimentin, snail and slug, and decreased level of claudin-1 (Fig. 5A and D, Table S6), suggesting that fentanyl induces an EMT in ovarian cancer.

### Fentanyl acts on ovarian cancer in EGFR- and opioid receptor-dependent manner

**Fig. 6:**
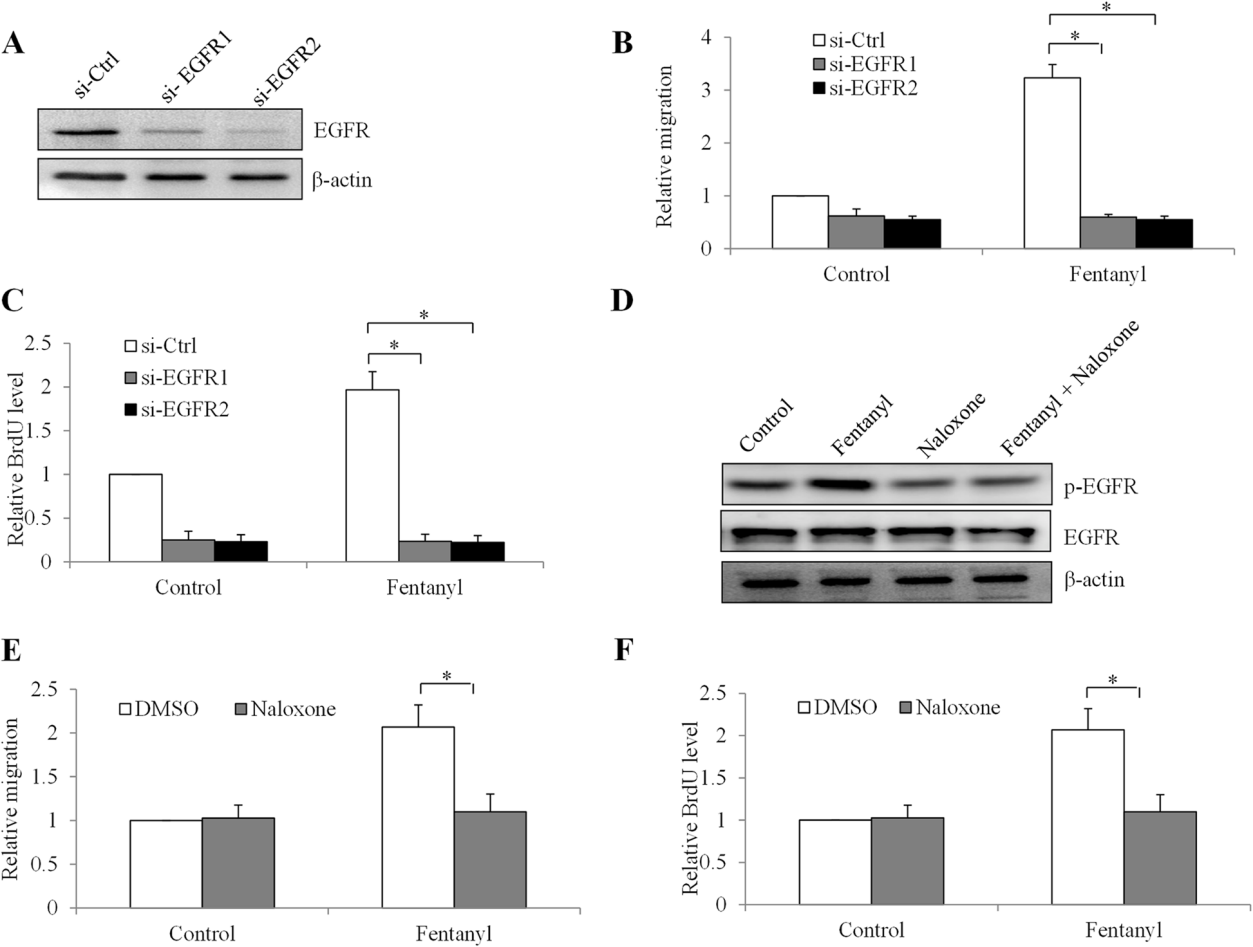
Fentanyl activates ovarian cancer cells via stimulating EGFR and in an opioid receptor-dependent manner. **A** Western blot of EGFR in SK-OV-3 cells after si-EGFR knockdown. EGFR depletion significantly reverses the effects of fentanyl in increasing proliferation (**B**) and migration (**C**) in SK-OV-3 cells. **D** Western blot of p-EGFR and EGFR in SK-OV-3 cells after in the presence of naloxone and fentanyl alone, or both. The blots were cut prior to hybridization with antibodies during blotting. Naloxone (10 μM) completely reverses the effects of fentanyl in increasing proliferation (**E**) and migration (**F**) in SK-OV-3 cells. *, *p* < 0.01, compared to control

To confirm the role of EGFR in fentanyl’s action, we knockdown EGFR in SK-OV-3 cells using two independent siRNA and validated their efficacy (Fig. 6A and Fig. S2). EGFR-depleted ovarian cancer cells were then treated with fentanyl, followed by migration and proliferation analysis. As expected, EGFR depletion alone decreased ovarian cancer migration and proliferation. Of note, fentanyl was ineffective in increasing migration and proliferation in EGFR-depleted cells (Fig. 6B and C, Table S7 and S8). This result suggests that EGFR is required for fentanyl’s action in ovarian cancer. Naloxone is a competitive opioid antagonist with a higher affinity for the μ receptor [22]. To determine whether μ receptor is involved in fentanyl-induced EGFR activation, we examined the phosphorylation of EGFR in the presence of both fentanyl and naloxone. We found that naloxone alone did not influence p-EGFR level. However, naloxone completely reversed the increased p-EGFR by fentanyl (Fig. 6D and Fig. S3). As expected, naloxone also completely reversed the increased migration and proliferation by fentanyl (Fig. 6E and F, Table S9 and S10). This indicates that μ receptor plays an important role in EGFR activation-induced by fentanyl in ovarian cancer cells.

## Discussion

Fentanyl has become the most often used opioid for intraoperative analgesia and management of chronic pain of all forms of cancer [23]. Using pre-clinical models, a number of studies have highlighted the direct effect of fentanyl on cancer cells, but both stimulatory and inhibitory effects were observed [12, 14, 15, 24, 25]. In addition, there is no universal underlying mechanism of the action of fentanyl on cancer cells, and via either opioid receptor-dependent or -independent mechanisms such as inhibition of Ets-1 and HDAC5 [13, 26], and activation of wnt/β-catenin [15]. We speculate that the effect and underlying mechanisms of fentanyl on cancer might be dependent on specific cancer types. A comprehensive understanding of potential implication of fentanyl in tumor biology is important as it might influence the long-term outcome of cancer patients. In this work, we demonstrate that fentanyl enhances ovarian cancer cell biological functions and reverses chemotherapy-induced apoptosis through activation of EGFR, and furthermore that this is associated with opioid receptor.

Three human ovarian cancer cell lines, SK-OV-3, TOV-21G and SW626, we selected for demonstration of the biological effects of fentanyl model ovarian cancer harbouring different histological and genetic profiling. Fentanyl at 100 to 400 nM which are equivalent to 0.033–0.13 µg/ml are used in our study. Using multiple cellular assays, we show that fentanyl at clinical concentrations promotes ovarian cancer growth and migration without affecting survival. Fentanyl displays anti-cancer activities in leukemia and pancreatic cancer [12, 14, 25] but pro-cancer activities in breast cancer and lung cancer [15, 21]. We add ovarian cancer to the list of cancers that their growth and invasion can be stimulated by fentanyl. Most studies investigated the effect of fentanyl alone in cancer whereas few studies analysed the combinatory effects of fentanyl with anti-cancer drugs. We show that the combination of fentanyl with cisplatin and paclitaxel is antagonistic. This is significant as this finding suggests that fentanyl might alleviate chemotherapy’s efficacy in ovarian cancer patients if both drugs are given concurrently. However, Dai et al. showed that fentanyl enhanced the tyrosine kinase inhibitor’s efficacy in leukemia [25] and Nomura et al. found that fentanyl did not affect 5-fluorouracil’s efficacy in colon cancer cells [27]. We noted that fentanyl inhibits leukemia cells and augments tyrosine kinase inhibitor’s efficacy; fentanyl stimulates ovarian cancer cells and abolishes chemotherapy’s efficacy; fentanyl neither stimulates nor inhibits colon cancer cells and does not affect chemotherapy’s efficacy. Fentanyl has a low therapeutic blood concentration of approximately 0.6 to 3 ng/ml for analgesia [28]. Fentanyl at 100 to 400 nM is used in our study for *in vitro* analysis which is equivalent to 0.033–0.13 µg/ml (using nM to ng/ml convention formula). Although these concentrations of fentanyl are higher than achievable in current therapeutic use, our work provides a proof-of-concept that fentanyl has pro-cancer activity in ovarian cancer. In addition, our data demonstrates that fentanyl at 40 µg/kg significantly promotes ovarian cancer growth in mice. Similar dose has been used in clinics for patients under some particular conditions [29, 30].

A significant finding of our work, in agreement with the previous reports [9, 10], is that the molecular mechanism of fentanyl on ovarian cancer cells is via activating EGFR. We demonstrated that fentanyl increased phosphorylation of EGFR and activated its downstream signalling, MEK/ERK and PI3K/Akt. Aberrant activation of PI3K/Akt plays an important role in ovarian cancer tumorigenesis and progression via regulating molecules involved in proliferation, survival, transcription and angiogenesis [31]. The ability of fentanyl in activating PI3K/Akt correlates well with its pro-proliferative and pro-survival effects in ovarian cancer cells. EGFR depletion abolishes the effects of fentanyl in ovarian cancer, confirming that EGFR is the target of fentanyl. It is known that EGFR is overexpressed in cancer cells but not normal cells [32]. We speculate that the less activity of fentanyl l on normal cells compared with ovarian cancer cells might correlate with differential expression level of EGFR in normal and tumor cells. We further demonstrate that fentanyl induces EGFR via acting on the endogenous µ opioid-receptor. G protein-coupled receptors (GPCRs) and receptor tyrosine kinases (RTKs) form heterocomplexes and trigger intracellular signalling and cellular responses. The transactivation of RTKs by GPCRs was demonstrated for EGFR, PDGFR and insulin-like growth factor receptor (IGFR) [33]. Although fentanyl has been shown to increase phosphorylation of PDGFR-β signaling in a diabetic model [34], fentanyl does not affect PDGFR phosphorylation in ovarian cancer cells. Our findings demonstrate the specific activation of EGFR by fentanyl via µ opioid-receptor. The induction of EMT by fentanyl observed in our studies is also well correlated with the previous work that EGFR cooperates signal transducer to induce EMT in cancer cells [35].

Very few studies reported effect of fentanyl on clinical outcomes of ovarian cancer patients. Lin et al. conducted a small retrospective study on the potential effects of epidural or general anaesthesia (GA)/opioid anaesthesia on prognosis of ovarian cancer. Their results suggested that ovarian cancer patients who received GA and intravenous fentanyl analgesia have worse mortality rate than those who received epidural analgesia [36]. This is supported by our pre-clinical findings that fentanyl promotes ovarian cancer cell biological activities. Based on findings in patients (*n* > 900) undergoing surgery for non-small lung cell cancer, there was a significant association between high intraoperative fentanyl dose and decreased overall survival as well as recurrence-free survival [37]. Notably, there was no association between intraoperative fentanyl dose and recurrence-free survival or overall survival in colorectal cancer patients (*n* > 1600) [38]. We speculate that the reason behind this might be due to cancer type specificity or the wildly varying nature of each study. The hypothesis should be tested in prospective randomized-controlled trials.

## Conclusion

Our work is the first to demonstrate that fentanyl at clinical achievable concentrations stimulates ovarian cancer cell growth and migration, and acts antagonistically with chemotherapy. The stimulatory effects of fentanyl in ovarian cancer cells are attributed to its ability in activating EGFR, and this is associated with µ opioid-receptor. Our pre-clinical findings contribute a better understanding on all the possible effect of fentanyl in cancer. Our work might accelerate the prospective, randomized-controlled trials on fentanyl’s effect on the outcome of ovarian cancer patients.

## Supplementary Information

**Additional file 1:** **Table S1. **Average and standard derivation (SD) value of migration in fentanyl-treated ovarian cancer cells. Results were presented as relative to control. Control was set as 1 in three independent experiments, thus SD of control is 0. **Table S2. **Average and standard derivation (SD) value of proliferation in fentanyl-treated ovarian cancer cells. Results were presented as relative to control. Control was set as 1 in three independent experiments, thus SD of control is 0. **Table S3. **Average and standard derivation (SD) value of apoptosis in fentanyl-treated ovarian cancer cells. Results were presented as relative to control. Control was set as 1 in three independent experiments, thus SD of control is 0. **Table S4. **Average and standard derivation (SD) value of apoptosis in drug-treated ovarian cancer cells. Results were presented as relative to control. Control was set as 1 in three independent experiments, thus SD of control is 0. **Table S5. **Average and standard derivation (SD) value of WB density in fentanyl-treated ovarian cancer cells. Results were presented as relative to control. Control was set as 1 in three independent experiments, thus SD of control is 0. **Table S6. **Average and standard derivation (SD) value of WB density in fentanyl-treated ovarian cancer cells. Results were presented as relative to control. Control was set as 1 in three independent experiments, thus SD of control is 0.** Table S7. **Average and standard derivation (SD) value of migration in siEGFR-treated ovarian cancer cells. Results were presented as relative to control (si-Ctrl). Control (si-Ctrl) was set as 1 in three independent experiments, thus SD of control is 0.** Table S8. **Average and standard derivation (SD) value of proliferation in siEGFR-treated ovarian cancer cells. Results were presented as relative to control (si-Ctrl). Control (si-Ctrl) was set as 1 in three independent experiments, thus SD of control is 0.** Table S9.** Average and standard derivation (SD) value of migration in drug-treated ovarian cancer cells. Results were presented as relative to control (DMSO). Control (DMSO) was set as 1 in three independent experiments, thus SD of control is 0.** Table S10. **Average and standard derivation (SD) value of proliferation in drug-treated ovarian cancer cells. Results were presented as relative to control (DMSO). Control (DMSO) was set as 1 in three independent experiments, thus SD of control is 0. **Fig. S1.** Uncropped western blot images for Fig. 4A: p-PDGFR-β, PDGFR-β, p-EGFR, EGFR, p-ERK, ERK, p-90RSK, 90RSK, p-Akt, Akt, Vimentin, Snail, Slug, Claudin-1 and β-actin. Three independent experiments were included. MW: molecular weight; WB: western blotting. The blots were cut prior to hybridisation with antibodies during blotting. **Fig. S2.** Uncropped western blot images for Fig. 5A. EGFR and β-actin. Three independent experiments were included. MW: molecular weight; WB: western blotting. The blots were cut prior to hybridisation with antibodies during blotting. **Fig. S3.** Uncropped western blot images for Fig. 5D. p-EGFR, EGFR and β-actin. Three independent experiments were included. MW: molecular weight; WB: western blotting. The blots were cut prior to hybridisation with antibodies during blotting.

## Data Availability

The datasets used and/or analysed during the current study available from the corresponding author on reasonable request.

## Abbreviations

EGFR: Epidermal growth factor receptor
GPCR: G protein coupled receptor
DMSO: Dimethyl sulfoxide
CI: Combination index
BrdU: Bromodeoxyuridine/5-bromo-2'-deoxyuridine
SDS-PAGE: Sodium dodecyl sulfate–polyacrylamide
ANOVA: Analysis of variance
RTKs: Receptor tyrosine kinases
