# Supplementary material for: Fentanyl activates ovarian cancer and alleviates chemotherapy-induced toxicity via opioid receptor-dependent activation of EGFR
Source: BMC Anesthesiol. 2022 Aug 23;22:268. doi: 10.1186/s12871-022-01812-4 (PMC9396929; doi:10.1186/s12871-022-01812-4)
Supplement: Supplementary file 1 — Additional file 1: Table S1. Average and standard derivation (SD) value of migration in fentanyl-treated ovarian cancer cells. Results were presented as relative to control. Control was set as 1 in three independent experiments, thus SD of control is 0. Table S2. Average and standard derivation (SD) value of proliferation in fentanyl-treated ovarian cancer cells. Results were presented as relative to control. Control was set as 1 in three independent experiments, thus SD of control is 0. Table S3. Average and standard derivation (SD) value of apoptosis in fentanyl-treated ovarian cancer cells. Results were presented as relative to control. Control was set as 1 in three independent experiments, thus SD of control is 0. Table S4. Average and standard derivation (SD) value of apoptosis in drug-treated ovarian cancer cells. Results were presented as relative to control. Control was set as 1 in three independent experiments, thus SD of control is 0. Table S5. Average and standard derivation (SD) value of WB density in fentanyl-treated ovarian cancer cells. Results were presented as relative to control. Control was set as 1 in three independent experiments, thus SD of control is 0. Table S6. Average and standard derivation (SD) value of WB density in fentanyl-treated ovarian cancer cells. Results were presented as relative to control. Control was set as 1 in three independent experiments, thus SD of control is 0. Table S7. Average and standard derivation (SD) value of migration in siEGFR-treated ovarian cancer cells. Results were presented as relative to control (si-Ctrl). Control (si-Ctrl) was set as 1 in three independent experiments, thus SD of control is 0. Table S8. Average and standard derivation (SD) value of proliferation in siEGFR-treated ovarian cancer cells. Results were presented as relative to control (si-Ctrl). Control (si-Ctrl) was set as 1 in three independent experiments, thus SD of control is 0. Table S9. Average and standard derivation (SD) value of mig [file 12871_2022_1812_MOESM1_ESM.doc]

**Fentanyl activates ovarian cancer and alleviates chemotherapy-induced toxicity via opioid receptor-dependent activation of EGFR**

**Table S1: Average and standard derivation (SD) value of migration in fentanyl-treated ovarian cancer cells.** Results were presented as relative to control. Control was set as 1 in three independent experiments, thus SD of control is 0.

**Table S2: Average and standard derivation (SD) value of proliferation in fentanyl-treated ovarian cancer cells.** Results were presented as relative to control. Control was set as 1 in three independent experiments, thus SD of control is 0.

**Table S3: Average and standard derivation (SD) value of apoptosis in fentanyl-treated ovarian cancer cells.** Results were presented as relative to control. Control was set as 1 in three independent experiments, thus SD of control is 0.

**Table S4: Average and standard derivation (SD) value of apoptosis in drug-treated ovarian cancer cells.** Results were presented as relative to control. Control was set as 1 in three independent experiments, thus SD of control is 0.

**Table S5: Average and standard derivation (SD) value of WB density in fentanyl-treated ovarian cancer cells.** Results were presented as relative to control. Control was set as 1 in three independent experiments, thus SD of control is 0.

**Table S6: Average and standard derivation (SD) value of WB density in fentanyl-treated ovarian cancer cells.** Results were presented as relative to control. Control was set as 1 in three independent experiments, thus SD of control is 0.

**Table S7: Average and standard derivation (SD) value of migration in siEGFR-treated ovarian cancer cells.** Results were presented as relative to control (si-Ctrl). Control (si-Ctrl) was set as 1 in three independent experiments, thus SD of control is 0.

**Table S8: Average and standard derivation (SD) value of proliferation in siEGFR-treated ovarian cancer cells.** Results were presented as relative to control (si-Ctrl). Control (si-Ctrl) was set as 1 in three independent experiments, thus SD of control is 0.

**Table S9: Average and standard derivation (SD) value of migration in drug-treated ovarian cancer cells.** Results were presented as relative to control (DMSO). Control (DMSO) was set as 1 in three independent experiments, thus SD of control is 0.

**Table S10: Average and standard derivation (SD) value of proliferation in drug-treated ovarian cancer cells.** Results were presented as relative to control (DMSO). Control (DMSO) was set as 1 in three independent experiments, thus SD of control is 0.

**Fig. S1: Uncropped western blot images for Fig. 4A:** p-PDGFR-β, PDGFR-β, p-EGFR, EGFR, p-ERK, ERK, p-90RSK, 90RSK, p-Akt, Akt, Vimentin, Snail, Slug, Claudin-1 and β-actin. Three independent experiments were included. MW: molecular weight; WB: western blotting. The blots were cut prior to hybridisation with antibodies during blotting.

**Fig. S2: Uncropped western blot images for Fig. 5A:** EGFR and β-actin. Three independent experiments were included. MW: molecular weight; WB: western blotting. The blots were cut prior to hybridisation with antibodies during blotting.

**Fig. S3: Uncropped western blot images for Fig. 5D:** p-EGFR, EGFR and β-actin. Three independent experiments were included. MW: molecular weight; WB: western blotting. The blots were cut prior to hybridisation with antibodies during blotting.
